# Multivalent cations interactions with fluoroquinolones or tetracyclines: A cross-sectional study

**DOI:** 10.1016/j.sjbs.2021.07.065

**Published:** 2021-07-30

**Authors:** Khalid Eljaaly, Asalah Helal, Tamather Almandeel, Rawan Algarni, Samah Alshehri

**Affiliations:** Faculty of Pharmacy, King Abdulaziz University, Jeddah, Saudi Arabia

**Keywords:** Fluoroquinolone, Quinolone, Tetracycline, Mineral, Complexation, Interaction

## Abstract

**Introduction:**

Oral fluoroquinolones and tetracyclines are known to interact with divalent or trivalent cation-containing compounds (DTCCs) via chelation. The objective of this study is to describe the prevalence of these drug-drug interactions (DDIs) in an inpatient setting.

**Methods:**

A cross-sectional study of prospectively collected data were conducted at an academic tertiary care hospital. We included hospitalized adults who were receiving oral fluoroquinolones or tetracyclines with DTCCs in 2019. Our hospital uses electronic health records for medication ordering and handwritten medication administration records (MARs). The primary study outcome was the percentage of simultaneous administration of fluoroquinolones or tetracyclines with DTCCs, and the secondary outcome was the percentage of inappropriate separation time.

**Results:**

Among patients who received oral fluoroquinolones or tetracyclines, 47 patients (26.6%) were co-administered DTCCs and included in this study. Ciprofloxacin (n = 29; 61.7%) was the most commonly interacting antibiotic, followed by moxifloxacin (n = 12; 25.5%) and doxycycline (n = 6; 12.8%). The interacting DTCCs included iron-containing products and calcium-containing products, and half of the patients (n = 24; 51%) received DTCCs once daily. Most patients (n = 35; 74.5%) were found to receive oral fluoroquinolones or tetracyclines at the same time as DTCCs, while one (2.1%) received inappropriately separated DTCCs.

**Conclusions:**

Despite being a very known contraindicated DDI, the prevalence of simultaneous co-administration of oral fluoroquinolones or tetracyclines with polyvalent cations was extremely high in a hospital with handwritten MARs. Antimicrobial stewardship programs should target this DDI, and future studies should evaluate the impact of different practical solutions to this problem in different clinical settings.

## 1 Introduction

Oral fluoroquinolones and tetracyclines are widely prescribed antibiotics, partially because of their relative activity against Gram-positive bacteria, Gram-negative bacteria, and atypical bacteria (Appelbaum and Hunter, 2000, Eljaaly et al., 2017, Smilack, 1999). Fluoroquinolones have additional activity against mycobacteria, and some are also active against anaerobes (Appelbaum and Hunter, 2000, Eljaaly et al., 2017). Fluoroquinolones and tetracyclines share some similar side effects such as gastrointestinal and photosensitivity reactions, but each class has its unique side effects such as QTc prolongation and tendon rupture with fluoroquinolones or esophagitis with tetracyclines (Appelbaum and Hunter, 2000, Eljaaly et al., 2017, Eljaaly et al., 2019, Eljaaly et al., 2020, Eljaaly et al., 2021). Both fluoroquinolones and tetracyclines are available orally and some as intravenous formulations. Only when given orally, drug-drug interactions (DDIs) due to chelation can occur between fluoroquinolones or tetracyclines and divalent or trivalent cation-containing compounds (DTCCs) such as iron, calcium, zinc, magnesium, and aluminum. This chelation leads to the formation of an insoluble complex compound that is poorly absorbed from the gastrointestinal tract (D'arcy and Mcelnay, 1987, Pitman et al., 2019). The mean area under the concentration–time curve of ciprofloxacin is reduced by up to 42% when co-administered with calcium, and by up to 64% when co-administered with iron preparations (Pitman et al., 2019). Another antibiotic that also can cause chelation with DTCCs but not available at our institution and might be more commonly used in pediatrics is cefdinir (Eljaaly and Alshehri, 2020).

Co-administration at the same time and inappropriate separation may lead to a clinically significant decrease in antibiotic bioavailability resulting in an increase in bacterial resistance and treatment failure (D'arcy and Mcelnay, 1987, Pitman et al., 2019, Depestel et al., 2007). Spacing out the administration times is a common recommendation to avoid or minimize this DDI and to enable independent absorption for each drug by avoiding gastrointestinal chelation (Pitman et al., 2019, Depestel et al., 2007). However, inappropriate administration of these antibiotics with DTCCs has been reported in hospitalized patients despite being a well-known DDI and recommendations to avoid it (Pitman et al., 2019). The purpose of this study is to examine the prevalence of the simultaneous administration and inappropriate separation time between oral fluoroquinolones or tetracyclines and oral DTCCs in patients admitted at a hospital with a handwritten medication administration record (MAR).

## 2 Methods

### 2.1 Study design and data collection

This cross-sectional study of prospectively collected data were conducted at an academic tertiary care medical center in Jeddah, Saudi Arabia. It was approved by the research ethics committee. Using the hospital electronic health records (EHRs), we screened hospitalized patients 18 years of age or older who were receiving oral fluoroquinolones available at the hospital (ciprofloxacin and moxifloxacin) or tetracyclines (doxycycline) during the year 2019. Those receiving these antibiotics with any DTCC were included in this study. Our hospital uses EHRs for medication ordering and verification, and a handwritten MAR. Collected patient data included: age, sex, total oral drugs given to the patient, hospital unit, names of the prescribed antibiotic and DTCC, frequency of administration, administration times, and the total number of medications received.

### 2.2 Study outcomes and data analysis

The primary study outcome was the percentage of patients who received oral fluoroquinolones or tetracyclines at the same time as DTCCs. The secondary outcome was the percentage of patients who received these antibiotics with inappropriately separated DTCCs. With fluoroquinolones, the separation time was considered appropriate if ciprofloxacin was administered two hours before or six hours after DTCCs, and if moxifloxacin was administered four hours before or eight hours after DTCCs except calcium as it does not need separation. The separation time with doxycycline was considered appropriate if it was administered at least one to two hours before aluminum, calcium, or magnesium-containing products, and if iron was administered not less than three hours before or two hours after doxycycline (Lexi-Drugs, xxxx, Micromedex Solutions, xxxx). Descriptive statistics were used to summarize the data including mean ± standard deviation (SD) for continuous data and frequency (percentage) for categorical data. The data were analyzed using the SPSS software, version 24 (IBM, Chicago, IL, United States).

## 3 Results

Out of 177 screened patients who received oral fluoroquinolones or tetracyclines, 47 patients were enrolled in this study and the remaining were excluded due to not receiving any DTCC. The patient characteristics and study outcomes are presented in Table 1. The average age of patients was 51.5 years, and they received an average of eight oral drugs. Medical wards were the most frequent location of those patients (n = 29; 61.7%). The most commonly interacting antibiotic was ciprofloxacin (n = 29; 61.7%), followed by moxifloxacin (n = 12; 25.5%) and doxycycline (n = 6; 12.8%). The interacting DTCCs were iron-containing products alone (n = 28; 59.6%), calcium-containing products (n = 15; 31.9%), or both (n = 4; 8.5%). Half of the patients (n = 24; 51%) received the antibiotics twice daily, about half received multiple daily doses of calcium (n = 11; 57.9% of patients receiving calcium), and more patients received a single daily dose than multiple doses of iron (n = 21; 65.6% of patients receiving iron). The majority of patients (n = 35; 74.5%) were found to receive oral fluoroquinolones or tetracyclines at the same time as DTCCs, while one (2.1%) received inappropriately separated DTCCs.

**Table 1:** Patient characteristics and study outcomes.

Variable	Number of Patients (N = 47)
Patient demographics	
Age in years, mean ± SD	51.5 ± 18.8
Female	27 (57.4)
Total number of oral drugs, mean ± SD	8.0 ± 3.2
Hospital location	
Medical wards	29 (61.7)
Surgical wards	7 (14.9)
Isolation unit	8 (17.0)
Gynecology ward	3 (6.4)
Indication	
Urinary tract infection	18 (38.3)
Tuberculosis	9 (19.1)
Skin and soft tissue infection	6 (12.8)
Respiratory	4 (8.5)
Intra-abdominal infection	4 (8.5)
Other infections	6 (12.8)
Oral antibiotic	
Ciprofloxacin	29 (61.7)
Moxifloxacin	12 (25.5)
Doxycycline	6 (12.8)
Antibiotic administration daily frequency	
Once	23 (48.9)
Twice	24 (51.1)
Oral DTCC	
Iron-containing products	28 (59.6)
Calcium-containing products	15 (31.9)
Both iron- and calcium-containing products	4 (8.5)
Iron-containing products daily administration frequency	
Once	21 (44.7)
Twice	11 (23.4)
Calcium-containing products daily administration frequency	
Once	8 (17.0)
Twice	7 (14.9)
Three times	4 (8.5)
Study outcome	
Co-administration of antibiotics at the same time of DTCCs	35 (74.5)
Co-administration of antibiotics with inappropriately separated DTCCs	1 (2.1)

Data are presented as number (%) unless otherwise indicated. DTCC: divalent or trivalent cation-containing compound.

## 4 Discussion

Our study found that three-quarters of patients who received oral fluoroquinolones or tetracyclines with DTCCs were simultaneously co-administered in our institution. The remaining patients had appropriate separation time except one. Despite the availability of data about the need for separation of administration in package inserts and literature, this remains a significant problem in clinical practice with insufficient attention from healthcare providers (Pitman et al., 2019, Lexi-Drugs, xxxx, Micromedex Solutions, xxxx).

In our study, 57% of the patients are female and the mean age is 51.5 years. Since osteoporosis is more dominant in postmenopausal females, calcium supplementation is crucial (Klibanski et al., 2001). Iron‐deficiency anemia is highly prevalent in menstruating women worldwide, so daily iron supplementation is required in settings where anemia is a major problem with a prevalence of 40% or higher (World Health Organization, xxxx). Thus, the opportunity for concomitant administration and inappropriate separation time between oral fluoroquinolones or tetracyclines and DTCCs might be expected to be greater. The mean number of total oral drugs given to our patients was eight. Taking more concomitant medications may contribute to difficulty in appropriate administration for nursing staff. In the United States (U.S.), a case-control study showed that co-administration of levofloxacin with DTCCs was significantly associated with polypharmacy (Barton et al., 2005). Moreover, co-administration of DTCCs within two hours of levofloxacin occurred in 90.4% of levofloxacin courses, which is even a higher occurrence than our study. Yamanaka-Yuen et al. reported that pharmacists in their U.S. hospital evaluated these DDIs, and DTCCs were withdrawn and inappropriately spaced in 27% and 69% of cases, respectively (Yamanaka-Yuen and Cantu, 1990).

There are several potential management strategies to prevent or minimize this DDI (Pitman et al., 2019). Before a strategy is chosen by the healthcare team, it is important to assess the patient situation, preferences, cost, and availability of alternatives. Although some hospitals might have electronic alerts for DDIs, alert fatigue can occur resulting in missing DDIs. The most common and recommended strategy is spacing out dose administration, but this strategy may be less applicable for elderly patients with polypharmacy. Therefore, temporarily stopping or decreasing the frequency of DTCCs may improve the possibility of spacing out the time of dose administrations. In selected patients, oral antibiotics could be switched to the intravenous route, but this may be a source for catheter-associated infections and other complications, the higher cost compared to the oral route, and this may increase the length of hospital stay (D'arcy and Mcelnay, 1987). In a U.S. hospital, Lomaestro et al. reported that even though their hospital educated physicians, nurses, and pharmacists about these DDIs, the pharmacy electronic system showed warnings, and a warning was included in the label of fluoroquinolone bag sent to the hospital ward, these interventions did not appear to reduce these DDIs (Lomaestro and Lesar, 1994). A retrospective study in the U.S. delivered educational interventions by educating nursing staff on reducing this DDI (Depestel et al., 2007). Before the intervention, they found that oral antibiotics were given within 2 h of DTCCs in 29 out of 40 (72.5%) patients before starting the in-services education, a percentage similar to our study. An in-service involving multiple approaches was associated with a significantly reduced frequency of these DDIs to 4 out of 40 patients (10%; P < 0.001). This study considered the appropriate timing to be 2 h before or after administration of oral fluoroquinolones or tetracyclines with DTCCs, which is not fully in accordance with the currently available recommendations. When our study was designed, we followed the currently recommended separation time for each DTCC. It is worth mentioning that all the studies we found were located in the U.S. and were not recent. Our study could be the first study outside the U.S., and it is important to study these common DDIs in other countries. Even though our study was conducted in 2019, these DDIs are still extremely common and future studies should investigate the impact of several interventions that are practical, not difficult to implement, and sustainable.

Our study has some limitations. The study was performed at a single center and the sample size was not large despite including patients over one year. However, this was a descriptive study, not analytical, patients needed to be on both oral specific antibiotics and DTCCs to be included, and this sample size was comparable to the previous limited studies. Unlike previous studies, we prospectively collected the study data. Unlike our hospital which has a handwritten MAR, it might be easier for hospitals with electronic MAR to detect these DDIs and to change the time of administration by the pharmacy. On the contrary, it is difficult in hospitals with handwritten MARs for the pharmacy to detect these DDIs. The nursing could be included and educated by the pharmacists to avoid this problem as they are the ones administering medications. If the hospital has more clinical pharmacists, they should be able to reduce this DDI as part of their duties by routinely checking for potential DDIs. More studies are needed to evaluate the most appropriate interventions to minimize this type of DDI in different hospital settings.

In conclusion, our results demonstrate that DDIs between fluoroquinolones or tetracyclines and DTCCs can still occur frequently in the clinical settings, which indicates that there is insufficient awareness of some healthcare providers. Future studies should evaluate the success of different practical solutions to this problem.

## Declaration of Competing Interest

The authors declare that they have no known competing financial interests or personal relationships that could have appeared to influence the work reported in this paper.
